# Reference Ranges for Fetal Ventricular Global Longitudinal Strain (GLS) Using Bidimensional Speckle-Tracking Echocardiography: A Systematic Review

**DOI:** 10.3390/jcm15176536

**Published:** 2026-08-24

**Authors:** Danielle Bittencourt Sodré Barmpas, Maria de Fátima Monteiro Pereira Leite, Saint Clair Gomes Junior, Karla G. Camacho, Maria Virginia M. Peixoto, Heron Werner, Renato Augusto Moreira de Sá

**Affiliations:** 1 Fernandes Figueira National Institute of Women’s, Children’s and Adolescents’ Health (IFF/Fiocruz), Rio de Janeiro CEP 22250-020, Brazil; maria.leite@fiocruz.br (M.d.F.M.P.L.); saint.junior@fiocruz.br (S.C.G.J.); karla.camacho@fiocruz.br (K.G.C.);; 2 Fetal Medicine Department, Instituto de Estudos em Tecnologia da Saúde (IETECS), Rio de Janeiro CEP 22775-022, Brazil; 3 Department of Fetal Medicine, Biodesign Laboratory DASA/PUC, Campus Gávea, Rio de Janeiro CEP 22451-900, Brazil; 4 Hospital Universitário Pedro Ernesto, Rio de Janeiro State University (UERJ), Rio de Janeiro CEP 20551-030, Brazil; 5 Department of Obstetrics, Hospital de Cascais Dr. José de Almeida, 2755-009 Cascais, Portugal

**Keywords:** fetal heart, echocardiography, prenatal diagnosis, global longitudinal strain, speckle tracking echocardiography, reference values, reproducibility

## Abstract

**Background/Objectives:** The primary objective was to assess reference intervals for fetal Global Longitudinal Strain (GLS) using bidimensional speckle-tracking echocardiography (2D-STE), including only prospective studies specifically designed for this purpose. An additional objective was to evaluate studies’ methodological quality and reproducibility. **Methods:** This is a systematic review registered at PROSPERO (CRD420251038889). Five electronic databases (Web of Science, Scopus, MEDLINE/PubMed, EMBASE and LILACS) were searched, from inception to May 2025. Prospective studies specifically designed to establish 2D-STE GLS reference intervals in low-risk singleton pregnancies with normal fetuses were included. Data were independently extracted by two reviewers. Risk of bias was assessed using an adapted tool, including study design and statistical and reporting methods. **Results:** After the initial identification of 187 records, nine studies published between 2012 and 2025 were included. There was marked heterogeneity among the studies. Four articles achieved high-quality scores (>70%) and three of them reported similar left ventricular (LV) GLS at 24 weeks (−22%). Right ventricular absolute GLS values were slightly lower than LV numbers. Regression models for both ventricles showed GLS absolute values decreased with gestation across studies. The 2D-STE algorithm (endocardial versus myocardial) was the main source of discrepancy between studies. **Conclusions:** High-quality prospective studies show a consistent pattern of biventricular GLS variation with gestational age. However, technical heterogeneity, lack of standardization, operator subjectivity and vendor-specific algorithm differences currently limit the applicability of the method. Multicentric studies with large sample sizes, standardized protocols and artificial intelligence-assisted tools are needed to consolidate this technique.

## 1. Introduction

Fetal echocardiography has evolved from a simple anatomy check to assessing cardiac function. Methods were adapted from adult echocardiography; however, they present technical difficulties in the fetus, due to their strict angle dependency, variable fetal position, the small size of the fetal heart and maternal and fetal movements [1]. These limitations have an impact on the feasibility and reproducibility of the exam and require specialized and experienced personnel for the assessment. Bidimensional speckle-tracking (2D-STE) is widely used in adult and pediatric echocardiography and started being adapted for fetuses two decades ago [2]. It measures myocardial deformation throughout the cardiac cycle in a short clip of the heart. The analysis is fairly angle independent and less challenging to perform than standard cardiac function tests using M-mode and Doppler [3,4]. Additionally, 2D-STE can detect myocardial diastolic dysfunction and may become a valuable tool for understanding the impact of maternal and fetal disease on the developing heart. Apart from allowing the diagnosis of early subclinical cardiac dysfunction, a quantitative method may be used for risk stratification and monitoring of myocardial deterioration in high-risk pregnancies. This may help to predict infants’ morbimortality, define the best moment for delivery and plan postnatal treatment, ultimately improving perinatal outcomes [5]. 2D-STE seems to be a promising tool for quantitative fetal cardiac function assessment throughout several clinical settings, including maternal diabetes and hypertension, fetal growth restriction and cardiac anomalies [5,6,7,8]. However, the heterogeneity between the studies, contradictory reference range results and reproducibility issues currently limit the reliability and clinical use of the method.

Global longitudinal strain (GLS) is a measure of the relative longitudinal shortening of the ventricles and seems the most reproducible measurement acquirable via 2D-STE [9]. Although a standard method for obtaining the clips and performing the measurement has been clearly defined [3,8], prospective data on reference values are scarce and present substantial differences on results according to gestational age (GA), acquisition equipment and 2D-STE analysis software, as was found by a previous systematic review on the subject [7,10,11,12,13,14,15,16,17,18,19,20,21].

The aim of this study is to assess reference ranges for fetal left and right ventricle GLS using only prospective studies specifically designed for this purpose, according to gestational age and equipment/software. Additional objectives include evaluating the methodological quality of the studies reviewed, the reproducibility of their data and how studies’ characteristics may affect the reference intervals.

## 2. Methods

This systematic review of the literature was performed based on the PRISMA (Preferred Reporting Items for Systematic Reviews and Meta-Analyses) criteria (www.prisma-statement.org URL accessed on 20 February 2025).

The PICO structure was designed as follows: Population (P) of healthy fetuses from low-risk singleton pregnancies; Intervention or Exposition (I or E), left or right ventricle GLS measurements acquired by 2D-STE in studies specifically designed with the aim to establish normal reference values for GLS (nomograms); Comparator (C), gestational age (GA), ultrasound equipment, speckle-tracking software, ventricle (left, right); Outcome (O), reference ranges for the measurements of biventricular fetal GLS with analysis of the compliance with methodological requirements for nomogram elaboration. The protocol was fully registered at PROSPERO 2025 CRD420251038889.

### 2.1. Eligibility Criteria

Inclusion criteria were prospective observational studies (cross-sectional or longitudinal) designed to describe GLS reference intervals of left and right ventricles measured with 2D-STE. The population should include low or general risk pregnant women and their singleton, adequately grown for gestational age (AGA) fetuses with previous negative screening for genetic conditions, anomalies and cardiac defects.

Exclusion criteria were retrospective or case-control studies, reviews, case reports, letters, abstracts presented at conferences and editorials. Studies were also excluded if they included twins and fetuses with possible cardiac dysfunction, women with medical conditions or pregnancy complications (such as hypertension, diabetes or infections).

### 2.2. Search Strategy

The search strategy was performed in the following electronic databases: Web of Science, Scopus, MEDLINE (PubMed), EMBASE and LILACS. It included articles published from inception to May 2025, without language restrictions. The terms were applied both individually and in combination. The full search strategies for all databases and the excluded articles with reasons are available in the Supplemental Files S1 and S2.

### 2.3. Data Extraction

The bibliographic references were managed on the Covidence platform. The selection resulting from the database search was conducted in different stages by two out of three independent reviewers (K.G.C., D.B.S.B, M.d.F.M.P.L.). Duplicates were identified and excluded. Titles and abstracts were examined first, and then full texts. The reference lists of the studies were manually searched to identify further eligible articles.

### 2.4. Data Analysis

GLS is a measure of myocardial deformation and is reported as a negative result. For uniformity of data, and in line with the adult WASE guideline [22], all GLS percentile results are classified as absolute values in this paper. Therefore, the numbers are consistent with the myocardial deformation. The smaller absolute number is set as the 5th percentile, and the larger value as the 95th percentile. In studies that originally defined the 5th percentile as the arithmetically smallest (most negative) value, percentile limits were reordered by absolute number during data extraction to provide consistency with the other articles. Regression equations given by the authors were compared.

The included works were reference-range studies reporting gestational-age-dependent GLS on non-comparable scales, with frequently missing or inconsistent dispersion data and differing speckle-tracking algorithms. Thus, it was not possible to perform a quantitative meta-analysis and the estimation of statistical heterogeneity, through the I^2^ statistic, and a structured narrative synthesis was used.

### 2.5. Methodological Quality Assessment

Methodological quality and risk of bias were assessed using an adaptation of a comprehensive set of criteria specifically designed for reference range studies. It was originally designed by Conde-Agudelo for fetal biometric charts [23], and is divided into three domains: study design, statistical analysis and reporting methods. It was later modified for Doppler studies [24] and used in other systematic reviews [23,24,25,26,27,28]. The instrument comprises 24 quality criteria. One point was awarded for each item fulfilled, and this was then transformed into a percentage. The risk of bias was independently assessed by two researchers (K.G.C. and D.B.S.B.) and disagreements were resolved by consulting a third reviewer (M.d.F.M.P.L. or M.V.M.P.).

## 3. Results

**Figure 1:**
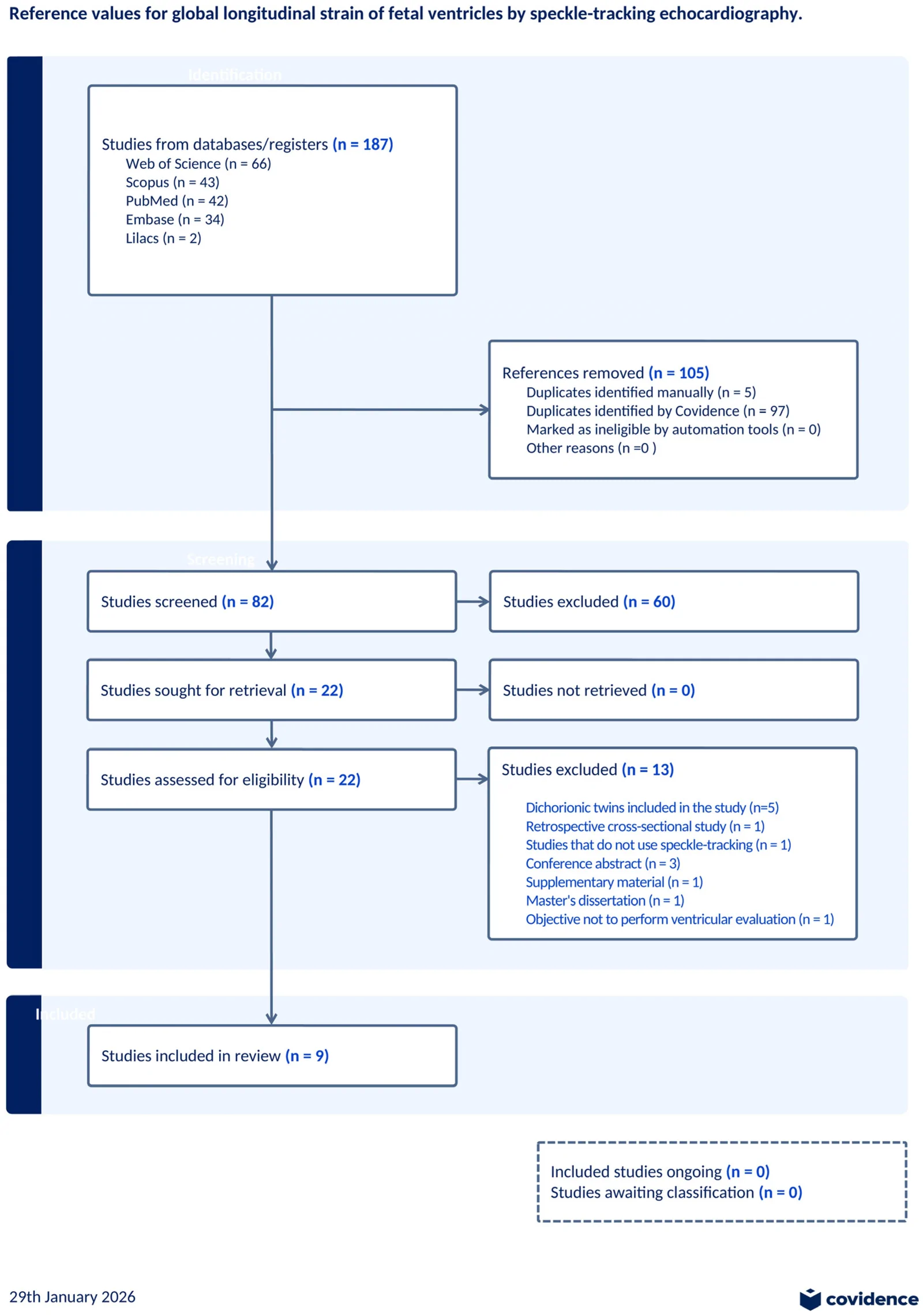
PRISMA flowchart summarizing the inclusion of studies. 2D-STE, bidimensional speckle-tracking echocardiography; GLS, global longitudinal strain.

Initially, 187 articles were found across five databases. After the initial removal of 102 duplicate records, the remaining titles and abstracts were screened for eligibility, and 22 articles were selected for full-text review. A further 13 studies were excluded after full-text assessment. Nine research papers published between 2012 and 2025 were selected for data extraction. The systematic review flowchart is presented in Figure 1.

**Figure 2:**
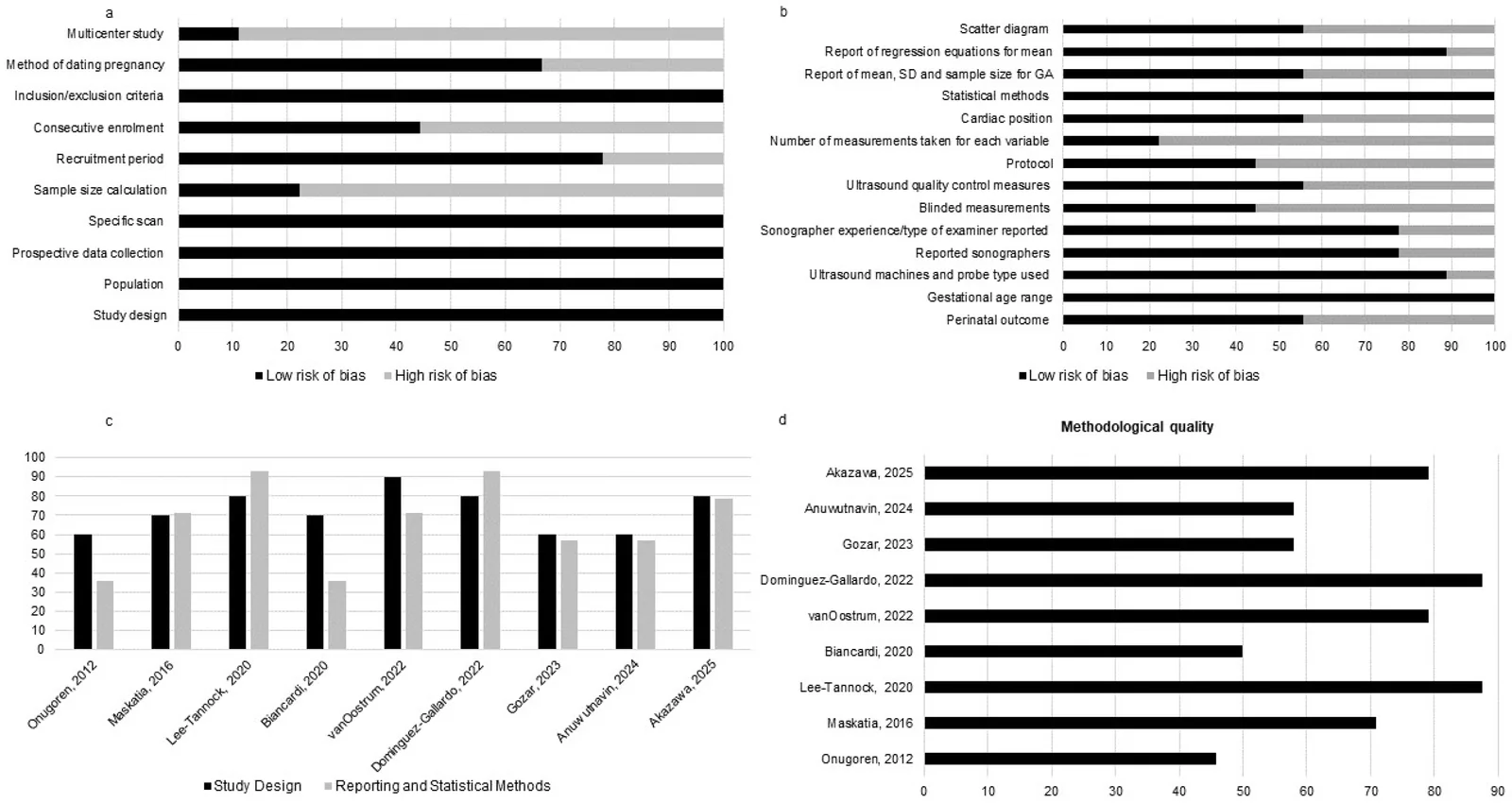
Methodological quality assessment of studies according to domain (study design; reporting and statistical methods), presented as a percentage of low risk of bias. The higher the percentage, the lower the risk of bias. (**a**) Score distribution of all studies for the study design domain; (**b**) score distribution of all studies in the reporting and statistical methods domain; (**c**) domain-specific quality score by author; (**d**) overall percentage of low risk of bias per study [7,10,11,12,13,16,17,19,20].

Figure 2 shows the percentage distribution of low risk of bias for all studies according to each criterion of study design and statistical methods, with final quality scores displayed both in total and by domain. No article scored less than 50% in the ‘study design’ domain; however, two studies fell below this threshold on the ‘reporting and statistical methods’ domain [11,19]. On the other hand, three research papers scored above 75% [7,12,16].

Research from nine different countries was assessed [7,10,11,12,13,16,17,19,20]. The characteristics of the studies are presented in Table 1. Four studies were cross-sectional [10,11,13,19] (4/9) and five were longitudinal [7,12,16,17,20]. Only two authors presented the sample size calculation [17,20]. Although the mean number of patients per study was 160 (59–513), two articles (2/9) included fewer than 100 fetuses, and only three clearly reported the sample size per gestational week [13,16,17]. Research was mainly performed at single centers (8/9) [10,11,12,13,16,17,19,20] and at university hospitals (6/9) [10,12,13,17,19,20]. Only three studies detailed maternal demographic characteristics [12,16,20]. The women were mostly white, non-smokers, had a mean age of 32 years old and had a mean body mass index (BMI) of 23. Half of the subjects were nulliparous, and fetal gender was equally divided. Two other studies reported only the patients’ ethnical background, having recruited exclusively South-East and East Asian patients (Thai and Japanese) [7,10]. The gestational age (GA) assessed ranged from 18 weeks to term (Table 1), with one exception, which included pregnancies before that period [19].

Most authors clearly described the inclusion criteria for healthy fetuses from low-risk pregnancies [7,10,11,12,13,16,19,20]. However, just four articles detailed the perinatal outcomes [12,16,17,20]. Regarding the fetal population, although all authors defined it as adequate for gestational age (AGA), only two-thirds of them (6/9) specified the pregnancy dating method used. Crown-rump length (CRL) measured by first trimester ultrasound was the most frequently employed, both as a single method [12,16,20] and as confirmation of the last menstrual period (LMP) [7,10,11]. Most research (7/9) assessed both ventricles [7,10,11,16,17,19,20], but two authors studied only the left one (Table 2) [12,13].

Cardiac clips were acquired using ultrasound equipment from four manufacturers and using distinct transducers, with frequencies ranging from 1 to 9 MHz. Furthermore, six vendor-specific 2D-STE software applications were used for the GLS measurements. In one study three different probes were used [19].

2D-STE analysis was performed by a pediatric cardiologist in four studies [7,13,20] and by a fetal medicine specialist in two [10,12]. A mixed team (cardiologist and fetologist) was reported in one article [17], and the information was lacking in the other two [11,19]. Less than half of the studies (4/9) reported operator blinding [7,16,17,20], while others either indicated no blinding [11] or did not report on it (4/9) [10,12,13,19].

Seventy-eight percent of the articles (7/9) described failure rates [7,10,11,12,16,19,20], which ranged from 2% to 40% (Table 2). The rates were higher on earlier studies and on the right ventricle [19,20]. The two studies which did not report failure rates used cardiac image quality as an inclusion or exclusion criterion [13,17]. The reasons for exam failure described throughout the articles were related to poor image quality. The limitations were either maternal (adiposity, movements), fetal (unfavorable position, movements) or technical (four-chamber view not obtained, low frame rate, low reproducibility due to GA below 20 weeks). Only five studies (5/9) [7,10,11,12,16] described the cardiac position at the exam and four reported quality control measures or a protocol for image acquisition and measurement technique [7,12,13,16]. Additionally, only two authors (2/9) reported performing three measurements per scan [12,16].

Reproducibility data are also detailed in Table 2. Different sample sizes were used to assess intraobserver variability (15 to 120) [7,11,12,13,16]. The intraobserver intraclass correlation coefficient (ICC) was used by three authors (3/9) [7,11,12], and Bland–Altman plots were employed in five studies [7,12,13,17,19]. One research group used the coefficient of variation of measurements to calculate intra- and interobserver variability [16]. Four authors employed more than one method for reproducibility assessment [7,12,13,17]. Interobserver ICC was calculated in four studies [7,12,13,17], using a mean of 33 patients (15 to 59). A single article detailed reproducibility according to the gestational age range, demonstrating lower reproducibility in earlier gestational ages [13].

Table 3 and Table 4 present GLS data on the left and right ventricles. Five different ultrasound (US) equipment brands and six 2D-STE analysis software were used [7,12,13,16,17,19,20]. All the studies reported high acquisition frame rates (over 60 Hz), with one exception that failed to present this information [11].

Two articles (2/9) found no variation in LV GLS with advancing GA [10,13], while the other seven reported a small decrease in absolute values [7,12,17,19,20]. GLS data were presented in weekly intervals by four authors (4/9) [7,12,16,20], while others reported results by GA range [13,17] or presented a single grouped result [10,11,19].

Right ventricle GLS was reported to be stable throughout the pregnancy by three studies (3/7) [10,11,16], while the remaining four found absolute numbers to decrease slightly (Table 4) [7,17,19,20].

**Figure 3:**
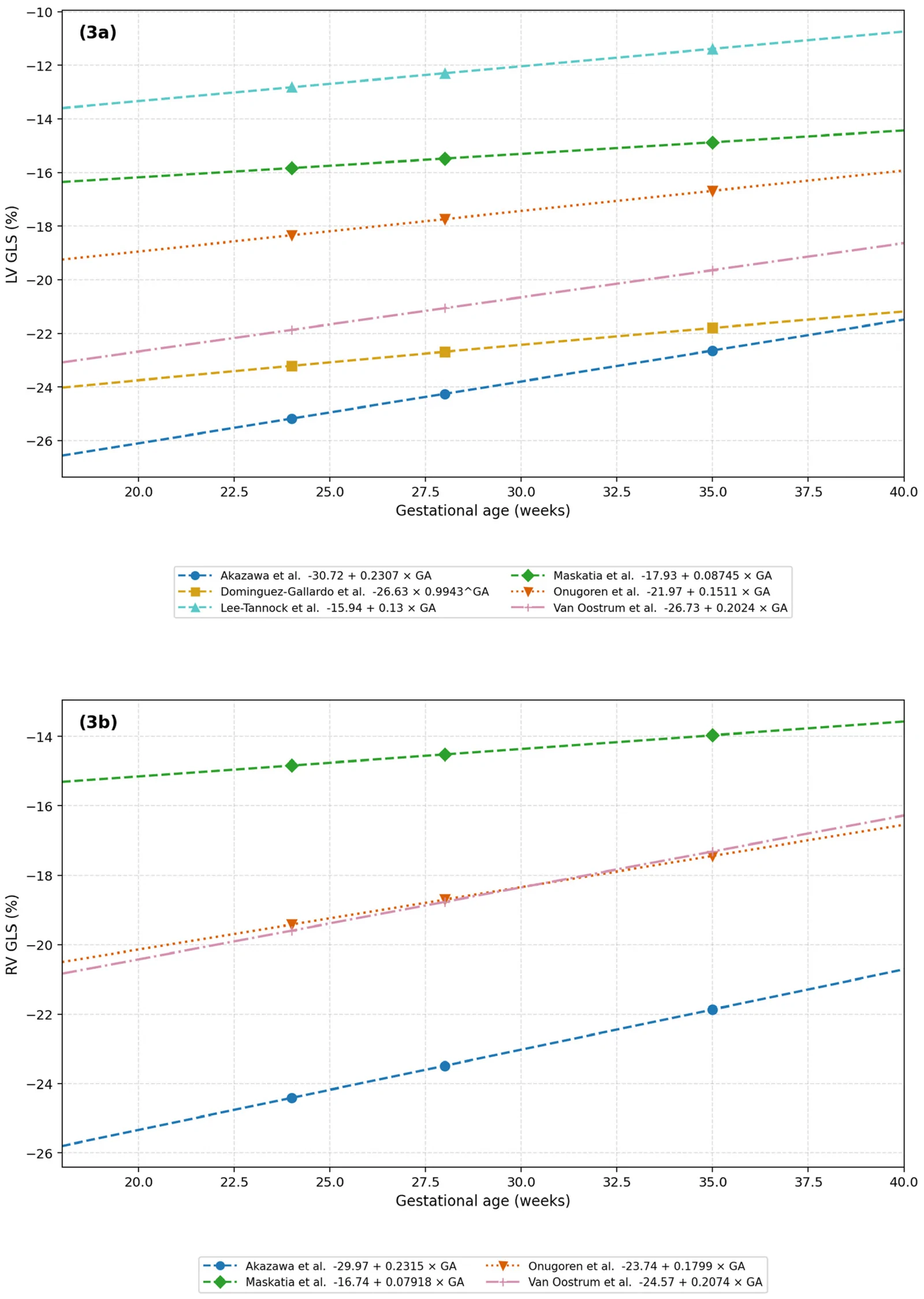
Comparative charts with published regression equations for fetal cardiac global longitudinal strain according to gestational age. (**a**) Left ventricle global longitudinal strain (LV GLS) with six published regression models [7,12,16,17,19,20]. (**b**) Right ventricle global longitudinal strain (RV GLS) with four regression models. GA, gestational age (weeks); GLS, global longitudinal strain; LV, left ventricle; RV, right ventricle [7,17,19,20].

All parameters of regression models were described for the left ventricle GLS by six studies [7,12,16,17,19,20] and for the RV by four [7,17,19,20]. Two comparative charts of ventricular curves were plotted to clarify the functional dynamics of the regression models (Figure 3). The graphs show subtle slopes and, despite some discrepancy in the absolute values, there is a remarkable similarity of the curves for both ventricles. Three of the four articles with the highest quality score found a similar mean LV GLS at 24 weeks (−22%) [7,12,20]. Another study with low risk of bias [16] found a lower LV GLS median (−12.87%). The main difference found between the groups was the use of endocardial or myocardial strain software (VVI).

A wide variation in standard deviation can be seen throughout the studies. Akazawa et al., with the largest sample size (513) [7], found a significantly lower SD (±2.7%) compared to the ±8.4% reported by Dominguez-Gallardo et al. (n = 152) [12]. Three articles describe SD variation across gestational weeks [7,13,17]. The 5th/95th percentiles are frequently used as ‘normality’ cut-offs in the clinical setting. Two works found a similar percentile range [7,12]. This can be seen at 24 weeks (−17%/−26%). A third reported a much broader interval (−9.8%/−33.9%) [20]. Although Lee-Tannock’s [16] GLS absolute values were different (−7.2/−18.5%), the reference interval was on the narrower range. Right ventricle GLS absolute values are generally slightly lower than those of the left. In the three highest-ranking articles, at 24 weeks, RV GLS mean ranged from −22.1% [7,20] to −14.9% [16]. The 5th/95th interval width pattern was similar in both ventricles; at 24 weeks, the RV thresholds were −18.2%/−27.2% [7]; −9.0%/−30.1% [20]; and −8.5%/−21.3% [16].

## 4. Discussion

Normative studies should combine accuracy and precision [29]. Accuracy results from an adequately purpose-designed study and recruiting a low-risk population, while precision depends on a large sample size and high reproducibility [29,30]. These were the principles behind this study. A previous systematic review assessed 2D-STE GLS reference ranges and found conflicting results [21]. However, many of the included studies were retrospective and not designed for reference interval construction, increasing the risk of bias. Consequently, our results were significantly different. The four studies with high-quality scores (>70%) [7,12,16,20] had similar regression models for biventricular GLS variation, with a slight decrease in absolute numbers with increasing GA.

Multiple sources of measurement variability were identified: subject-related (maternal/fetal movements, fetal lie, gestational age), technical (ultrasound equipment, speckle-tracking software), operator-related (image acquisition standardization, subjectivity, learning curve) and statistical methods (reproducibility assessment, data modelling).

### 4.1. Population

The most frequent methodological problem was the lack of sample size calculation [17,20]. Additionally, studies did not clearly report the number of fetuses per gestational week [7,11,19,20] and grouped results by GA ranges rather than weekly [10,11,17,31]. As GLS changes during the pregnancy, this may compromise the precision of GA-specific intervals [29,30,32]. Even among the four best-quality studies, the reference interval width changes remarkably [7,12,16,20]. Differences in SD and percentiles between studies are mainly attributed to variations in weekly sample sizes. Few studies detailed patients’ demographic characteristics [12,16,20], and most failed to describe pregnancy outcomes [7,10,11,19,33]. This raises concerns regarding the population’s low-risk status. Well documented perinatal data is fundamental to ensure that the inclusion criteria of healthy, adequate birthweight neonates were met [30]. Most subjects were white and there was no difference in GLS values between Caucasian and Asian groups [7,10,12,16]. However, no articles recruited large numbers of South Asian or black women, which represent a substantial part of the world’s population. Additionally, most studies were conducted in single centers, mainly university hospitals [10,12,13,17,19,20]. Such issues may compromise the generalizability of the findings.

### 4.2. Imaging

Ultrasound equipment and transducer type vary widely across articles. However, the impact of transducer frequency on final GLS results seems to be minor [34,35].

The variation in GLS means among the best-quality publications [7,12,16,20] could be explained by the 2D-STE software algorithm used (endocardial [7,12,20] vs. myocardial [16]). Previous publications comparing GLS results in fetal populations were unable to find this correlation [7,10]. However, in adults, endocardial strain had already been reported to be greater than myocardial strain [22]. WASE guidelines currently recommend using the same ultrasound equipment and speckle-tracking software for patient follow-up [22]. This should also apply to fetuses since recent results found disappointing reproducibility between 2D-STE software brands, regardless of the use of semi-automated techniques [36,37,38,39,40,41]. Vendor-specific GLS differences are well documented, but measurement reliability can also be affected by poor imaging, subjectivity in cycle selection and valve insertion marking, which can be partially explained by operator experience and the method’s long learning curve [5,36,37,41,42].

### 4.3. Operator-Related

Although technical guidelines for exam standardization were published [3,8], few studies reported the protocol used [7,8,12,16], cardiac position at examination [7,12,13,16] or ultrasound quality control measures [10,11,19,20]. This is particularly important due to ongoing debate about the impact of the insonation angle on GLS results, both in fetal and postnatal life [4,5,42,43,44]. Additionally, nearly half of the studies reported cardiac clip acquisition by multiple examiners [7,12,17,20] regardless of the recommendation for a single operator [29]. Other frequently missing data that may compromise accuracy are operator blinding [10,11,12,13,19] and performing at least three measurements per scan [7,10,11,13,17,19,20].

### 4.4. Statistical Methods

Failure rates dropped from 40% [19] to less than 8% [7] over 13 years, which may reflect technological or methodological improvement. However, the 8% figure refers only to strain analysis failure, excluding imaging limitations. If both factors are considered, the failure rate increases to 18%. A recent study performed in a clinical setting found that imaging limitation increased failure rates to nearly 50% [41]. Using suboptimal images or the inability to obtain a 4-chamber view as exclusion criteria [10,13,17] rather than describing them as failures introduces a reporting bias by overstating feasibility. The reproducibility of observational data validated by statistical methods (e.g., coefficient of variation, ICC, Bland–Altman plots) is fundamental for ensuring research reliability. Low reproducibility compromises reference thresholds and diagnostic accuracy in the clinical scenario [32]. The coefficient of variation is a reliable method for quantifying absolute measurement error in normative studies across gestational ages. Lee-Tannock et al. [16] used it, reporting good reproducibility. On the other hand, ICC is a relative measure influenced by between-subject variance in heterogeneous samples [45,46]. Its precision can be increased by calculating it according to gestational age [17] or adding a second evaluation, like Bland–Altman plots. Four authors used this approach [7,12,13,17], with two reporting good intra- and inter-observer ICC [7,12] (>0.75 with 95% CI) [46]. Five studies had incomplete reproducibility data [10,11,13,17,19], and one did not address it [20]. Lower ICC at 20–21 weeks of pregnancy [17] suggests that a smaller fetal size may limit exam reproducibility, which was corroborated by a later study [18]. The lack of standardization in the assessment methods between studies hampers the comparison of reproducibility and reliability. Additionally, two high-quality studies documented the unusual finding of intraobserver variability exceeding interobserver variability [12,16,45].

### 4.5. GLS Results

The lack of uniformity in methodology across articles impacts even the determination of percentiles. Most publications observe the WASE guidelines [22], using absolute values for classification. However, half (2/4) of the high-quality studies [7,20] diverge from this approach, defining the 5th percentile as the arithmetically smallest value (most negative). The inconsistent methodology is confusing and complicates the comparison of studies. The small decrease in biventricular absolute GLS values with GA reported by most studies [7,12,17,19,20] agrees with additional research not assessed by this review [15,47,48,49,50,51,52,53]. The articles that found LV GLS to be stable throughout pregnancy [10,13] had lower methodology scores (<60%). An additional strong finding was the consistency of LV GLS found in the second trimester across the three lowest bias papers [7,12,20] using different ultrasound equipment. The impact of sample size in measurement variability (SD) becomes evident in the single article describing both variables per GA [17]. Considering similar low-risk maternal-fetal populations across studies, differences in reference interval widths may be attributed to weekly sample size. Right ventricle GLS absolute measurements were slightly lower than LV, which can be explained by differences in the ventricular mechanics, as reported by other authors [18,54,55]. Among the three highest-scoring articles [10,11,16], one reported stable values across the pregnancy [16], while the other two found a small decrease in absolute numbers with advancing GA [7,20]. For the RV, this difference could not be attributed to sample size or risk of bias. On the other hand, the analysis of RV GLS regression models also revealed comparable curves [7,17,19,20]. The geometry of the right ventricle contributes to the exam’s technical difficulty, increasing failure rates and measurement variability and decreasing reproducibility.

### 4.6. Strengths and Limitations

This is the first systematic review to include only prospective studies specifically designed to create reference intervals. Its strength lies in the thorough objective evaluation of methodological quality and risk-of-bias using a tool covering study design, statistical analysis and reproducibility. Limitations included the inability to perform meta-analysis due to significant study heterogeneity, use of non-comparable scales (weekly values, gestational-age ranges, single vs. grouped results) and frequent lack of weekly sample sizes and standard deviations.

### 4.7. Future Research

Future research should minimize exam variability by using multicentric studies, large sample sizes across pregnancy with a minimum number of patients per week, standardized protocols and AI tools for image acquisition and assessment. Meta-analysis of individual patient data from high-quality studies and a mathematical conversion model between algorithm-specific results may be additional lines of investigation.

## 5. Conclusions

Bidimensional speckle-tracking GLS is a promising tool for the quantitative assessment of fetal cardiac function. However, currently, its clinical applicability is limited by the lack of a common reference interval, technical variability, lack of exam standardization, suboptimal images and examiner subjectivity. Reproducibility remains a key limitation: inconsistent use of the coefficient of variation, ICC and Bland–Altman analysis, incomplete reporting and reduced reliability at earlier gestational ages preclude comparison across studies and hinder clinical adoption. This review identified a common pattern of GLS variation with gestational age across the highest-quality studies. The main source of discrepancy between fetal GLS curves is the kind of 2D-STE software used. Future studies must address factors hampering the reliability of results before clinical implementation can be considered—specifically, the establishment of standardized acquisition and analysis protocols, unification of inter-vendor strain measurements, reproducibility meeting clinically acceptable thresholds and prospectively validated gestational-age-specific reference intervals. Regardless of the recent progress, until these benchmarks are met, the technique does not yet seem to be ready for the clinical scenario.

## Figures and Tables

**Table 1:** Characteristics of the included studies.

Authors, Year	Data Collection Period	Study Design	Inclusion Criteria	*n*	Gestational Age (Weeks + Days)
Onugoren et al., 2012 [19]	NR	Cross-sectional	Women without systemic diseases; singleton pregnancies 13 + 0 to 41 + 3 WG; AGA fetuses	137	13 to 41 + 3
Maskatia et al., 2016 [17]	October 2013 to May 2014	Longitudinal	Maternal BMI < 30; singleton pregnancy; acceptable fetal cardiac visualization; no cardiac abnormalities	59	20 to 37
Biancardi et al., 2020 [11]	May to November 2018	Cross-sectional	Maternal age ≥18 years; healthy mothers; singleton pregnancies 22 to 31 + 6 WG; healthy fetuses	156	22 to 31 + 6
Lee-Tannock et al., 2020 [16]	July 2015 to December 2017	Longitudinal	No maternal complications; singleton uncomplicated pregnancies 18–28 WG; AGA fetuses without anomalies	120	18 to 40
Domínguez-Gallardo et al., 2022 [12]	June 2018 to March 2020	Longitudinal	Healthy pregnant women; singleton pregnancies; no evidence of fetal structural cardiovascular disease	152	24 to 37
Van Oostrum et al., 2022 [20]	May 2018 to April 2019	Longitudinal	Healthy women; low-risk singleton pregnancy; normal anomaly scan in 2nd trimester; physiologically monitored pregnancies	124	18 to 41
Gozar et al., 2023 [13]	May 2020 to September 2022	Cross-sectional	AGA fetuses	103	22 to 40
Anuwutnavin et al., 2024 [10]	NR	Cross-sectional	Maternal age >18 years; uncomplicated singleton pregnancies 17–24 WG	79	17 to 24
Akazawa et al., 2025 [7]	May 2020 to November 2022	Longitudinal	Healthy women; uncomplicated singleton pregnancies 18–40 WG; healthy fetuses	513	18 to 40

AGA, adequate for gestational age; BMI, body mass index; NR, not reported; WG, weeks of gestation.

**Table 2:** Methodological and reproducibility data on global longitudinal strain (GLS) obtained by 2D-STE for both ventricles on studies designed to produce reference intervals.

Authors, Year	US Equipment	2D-STE Software	Ventricle Assessed	3 Measurements per Exam	Failure Rate (%)	Reproducibility *n*	Intraobserver ICC (95% CI)	Interobserver ICC (95% CI)
Onugoren et al., 2012 [19]	Acuson S 2000 (Siemens)	syngo VVI	Both	NR	40.4 (93/230)	68 LV 62 RV	NR	NA
Maskatia et al., 2016 [17]	Vivid I (GE)	EchoPAC	Both	NR	NR	59	NA	LV: 0.35 RV: 0.88
Biancardi et al., 2020 [11]	HM70 (Samsung)	2DSTE Samsung	Both	N	8.3 (15/181)	113 LV 118 RV	LV: 0.29 RV: 0.22	NR
Lee-Tannock et al., 2020 [16]	Acuson S 2000 (Siemens)	syngo VVI	Both	Y	3.9 (16/406)	120 (all GA)	CV: LV 8.3% RV 9.0%	CV: LV 6.0% RV 5.1%
Domínguez-Gallardo et al., 2022 [12]	Epiq W7 (Philips)	aCMQ-Qlab	LV only	Y	4.6 (20/435)	40	0.998 (0.99–0.99)	0.991 (0.984–0.995)
Van Oostrum et al., 2022 [20]	Epiq W7 (Philips)	2DCPA 2.1 (TomTec)	Both	NR	6.3 LV 10.4 RV	NR	NR	NR
Gozar et al., 2023 [13]	Epiq W7 (Philips)	aCMQ-Qlab	LV only	NA	NR	15	NA	0.85 (0.62–0.94)
Anuwutnavin et al., 2024 [10]	Voluson E10 (GE)	2D CPA TomTec	Both	N	7.0 (6/85)	NR	NR	NR
Akazawa et al., 2025 [7]	Voluson E10 (GE)	fetal HQ	Both	N	18.0 both 1.8 LV † 7.2 RV †	30	LV: 0.90 (0.81–0.95) RV: 0.87 (0.75–0.94)	LV: 0.91 (0.83–0.96) RV: 0.86 (0.74–0.93)

CV, coefficient of variation; CI, confidence interval; GA, gestational age; ICC, intraclass correlation coefficient; LV, left ventricle; n, number of fetuses; N, no; NA, not assessed; NR, not reported; RV, right ventricle; SD, standard deviation; US, ultrasound; Y, yes. † Failure on 2D-STE analysis of clips only.

**Table 3:** 2D-STE left ventricular global longitudinal strain: means, standard deviations, 5th and 95th percentiles and regression equations.

Authors, Year	US Equipment and Analysis Software	Transducer	*n*	Frame Rate (Fps)	GA Range (Weeks)	Mean GLS (%) †	SD/IQR	5th Centile	95th Centile
Onugoren et al., 2012 [19]	Acuson S 2000 (Siemens), syngo VVI	Linear 4–9 MHz; convex 2.5–6 MHz; volumetric 3–6.5 MHz	68	114 (57–241)	13 to 41 + 3	18.43	3.57	10.56	29.01
Maskatia et al., 2016 [17]	Vivid I (GE), EchoPAC	Matricial 5–6 MHz	59	152 (100–340)	20 to 37	19.61–21.13	3.71–2.90	—	—
Biancardi et al., 2020 [11]	HM70 (Samsung), 2DSTE Samsung	Pediatric sectorial 4.5 MHz, P3-8 (3–8 MHz)	150	NR	22 to 31 + 6	16.9	8.4	4.3	48.8
Lee-Tannock et al., 2020 [16]	Acuson S 2000 (Siemens), syngo VVI	Convex 3–8 MHz	120	94 (75–181)	18 to 40	Median 13.64–10.81	4.7 IQR	8.04–5.09	19.26–16.52
Domínguez-Gallardo et al., 2022 [12]	Epiq W7 (Philips), aCMQ-Qlab	Sectorial 9 MHz (C9-2)	152	106	24 to 37	22.0–20.2	8.4	17.73–16.45	26.74–24.81
Van Oostrum et al., 2022 [20]	Epiq W7 (Philips), 2DCPA 2.1 (TomTec)	Linear 9 MHz	124	83 (73–96)	18 to 41	23.08–18.43	—	10.35–7.07	35.82–29.79
Gozar et al., 2023 [13]	Epiq W7 (Philips), aCMQ-Qlab	NR	39	>80	22 to 40	15.59–14.80	4.84–3.80	—	—
Anuwutnavin et al., 2024 [10]	Voluson E10 (GE), 2D CPA TomTec	Convex C1-5-D, RAB6-D	79	98 ± 15	17 to 24	31.81	6.53	—	—
Akazawa et al., 2025 [7]	Voluson E10 (GE), fetal HQ	Matricial eM6C (2–7 MHz)	504	116 ± 15.9	18 to 39	27.3–21.0	4.3–5.4	21.3–17.3	35.5–24.8

† Absolute values. 2D-STE, bidimensional speckle-tracking echocardiography; fps, frames per second; GA, gestational age; GLS, global longitudinal strain; IQR, interquartile range; NR, not reported; SD, standard deviation; US, ultrasound. All data were directly extracted from the original studies. GLS values are expressed as absolute numbers, in accordance with the WASE guidelines [22]. The smaller absolute value corresponds to the 5th percentile and the larger to the 95th percentile. In studies [7,20] originally defining the 5th percentile as the arithmetically smallest (most negative) value, percentile limits were reordered by absolute magnitude during data extraction to ensure comparability across studies.

**Table 4:** 2D-STE right ventricular global longitudinal strain: means, standard deviations, 5th and 95th percentiles and regression equations.

Authors, Year	US Equipment and Analysis Software	Transducer	*n*	Frame Rate (Fps)	GA Range (Weeks)	Mean/Median GLS (%) †	SD/IQR	5th Centile	95th Centile
Onugoren et al., 2012 [19]	Acuson S 2000 (Siemens), syngo VVI	Linear 4–9 MHz; convex 2.5–6 MHz; volumetric 3–6.5 MHz	68	114 (57–241)	13 to 41 + 3	18.43	3.02	12.61	26.75
Maskatia et al., 2016 [17]	Vivid I (GE), EchoPAC	Matricial 5–6 MHz	59	152 (100–340)	20 to 37	18.82–19.54	3.13–2.56	—	—
Biancardi et al., 2020 [11]	HM70 (Samsung), 2DSTE Samsung	Pediatric sectorial 4.5 MHz, P3-8 (3–8 MHz)	155	NR	22 to 31 + 6	14.5	7.3	4.5	42.8
Lee-Tannock et al., 2020 [16]	Acuson S 2000 (Siemens), syngo VVI	Convex 3–8 MHz	120	94 (75–181)	18 to 40	Median 14.91	NR	8.55	21.31
Van Oostrum et al., 2022 [20]	Epiq W7 (Philips), 2DCPA 2.1 (TomTec)	Linear 9 MHz	124	83 (73–96)	18 to 41	20.84–16.07	NR °	10.25–5.24	31.43–26.89
Anuwutnavin et al., 2024 [10]	Voluson E10 (GE), 2D CPA TomTec	Convex C1-5-D, RAB6-D	79	98 ± 15	17 to 24	28.85	6.24	—	—
Akazawa et al., 2025 [7]	Voluson E10 (GE), fetal HQ	Matricial eM6C (2–7 MHz)	504	116 ± 15.9	18 to 39	27.1–21.0	5.1–3.3	19.2–18.7	35.1–23.3

† Absolute values. ° SD not available; only 5th–95th percentile ranges reported. 2D-STE, bidimensional speckle-tracking echocardiography; fps, frames per second; GA, gestational age; GLS, global longitudinal strain; IQR, interquartile range; NA, not assessed; NR, not reported; SD, standard deviation; US, ultrasound. All data were directly extracted from the original studies. GLS values are expressed as absolute numbers, in accordance with the WASE guideline [22]. The smaller absolute value corresponds to the 5th percentile and the larger to the 95th percentile. In studies [7,20] originally defining the 5th percentile as the arithmetically smallest (most negative) value, percentile limits were reordered by absolute magnitude during data extraction to ensure comparability across studies.

## Data Availability

The data that supports the findings of this study are available in the Supplementary Materials of this article.

## Supplementary Materials

The following supporting information can be downloaded at: https://www.mdpi.com/article/10.3390/jcm15176536/s1, PRISMA_2020_checklist; S1: Full search strategies for all databases; S2: Excluded articles with reasons; S3: Full list of methodological quality criteria.
