# Supplementary material for: Reference Ranges for Fetal Ventricular Global Longitudinal Strain (GLS) Using Bidimensional Speckle-Tracking Echocardiography: A Systematic Review
Source: J Clin Med. 2026 Aug 24;15(17):6536. doi: 10.3390/jcm15176536 (PMC13566355; doi:10.3390/jcm15176536)
Supplement: Supplementary file 1 [file jcm-15-06536-s001.zip › jcm-4423392-supplementary/S1_Full_Search_strategies_for_all_databases.pdf]

S1 – Full Search strategies for all databases.

(Web of Science = 66)
(Fetus OR Fetal* OR Fetus*) AND ("Global Longitudinal Strain" OR LV-GLS OR "Left Ventricular Global Longitudinal Strain" OR RV-GLS OR "Right Ventricular Global Longitudinal Strain" OR speckle-tracking OR "2D strain" OR "Longitudinal strain") AND ("reference values" OR "normal values" OR nomogram OR "reference range" OR "normal range") AND (echocardiography OR "2-D Echocardiography" OR "2D Echocardiography" OR "Cross-Sectional Echocardiography" OR "M-Mode Echocardiography" OR "Transthoracic Echocardiography" OR "Two-Dimensional Echocardiography" OR ultrasound) (Topic)

(Scopus = 43)
(INDEXTERMS (fetus) OR TITLE-ABS (fetal*) OR TITLE-ABS (fetus*)) AND (INDEXTERMS ("Global Longitudinal Strain") OR TITLE-ABS ("Global Longitudinal Strain") OR TITLE-ABS (lv-gls) OR TITLE-ABS ("Left Ventricular Global Longitudinal Strain") OR TITLE-ABS (rv-gls) OR TITLE-ABS ("Right Ventricular Global Longitudinal Strain") OR TITLE-ABS (speckle-tracking) OR TITLE-ABS ("2D strain") OR TITLE-ABS ("Longitudinal strain")) AND (INDEXTERMS ("reference values") OR TITLE-ABS ("reference values") OR TITLE-ABS ("normal values") OR TITLE-ABS (nomogram) OR TITLE-ABS ("reference range") OR TITLE-ABS ("normal range")) AND (INDEXTERMS (echocardiography) OR TITLE-ABS (echocardiography) OR TITLE-ABS ("2-D Echocardiography") OR TITLE-ABS ("2D Echocardiography") OR TITLE-ABS ("Cross-Sectional Echocardiography") OR TITLE-ABS ("M-Mode Echocardiography") OR TITLE-ABS ("Transthoracic Echocardiography") OR TITLE-ABS ("Two-Dimensional Echocardiography") OR TITLE-ABS (ultrasound))

(Medline via PubMed = 42)
("Fetus"[mh] OR Fetal*[tiab] OR Fetus*[tiab]) AND ("Global Longitudinal Strain"[mh] OR "Global Longitudinal Strain"[tiab] OR "LV-GLS"[tiab] OR "Left Ventricular Global Longitudinal Strain"[tiab] OR "RV-GLS"[tiab] OR "Right Ventricular Global Longitudinal Strain"[tiab] OR "speckle-tracking"[tiab] OR "2D strain"[tiab] OR "Longitudinal strain"[tiab]) AND ("reference values"[mh] OR "reference values"[tiab] OR "normal values"[tiab] OR "nomogram"[tiab] OR "reference range"[tiab] OR "normal range"[tiab]) AND ("echocardiography"[mh] OR "echocardiography"[tiab] OR "2-D Echocardiography"[tiab] OR "2D Echocardiography"[tiab] OR "Cross-Sectional Echocardiography"[tiab] OR "M-Mode Echocardiography"[tiab] OR "Transthoracic Echocardiography"[tiab] OR "Two-Dimensional Echocardiography"[tiab] OR "ultrasound"[tiab])

(Embase = 34)
('fetus'/exp OR fetal*:ti,ab OR fetus*:ti,ab) AND ('global longitudinal strain'/exp OR 'global longitudinal strain':ti,ab OR 'lv gls':ti,ab OR 'left ventricular global longitudinal strain':ti,ab OR 'rv gls':ti,ab OR 'right ventricular global longitudinal strain':ti,ab OR 'speckle tracking':ti,ab OR '2d strain':ti,ab OR 'longitudinal strain':ti,ab) AND ('reference values'/exp OR 'reference values':ti,ab OR 'normal values':ti,ab OR nomogram:ti,ab OR 'reference range':ti,ab OR 'normal range':ti,ab) AND ('echocardiography'/exp OR echocardiography:ti,ab OR '2-d echocardiography':ti,ab OR '2d echocardiography':ti,ab OR 'cross-sectional echocardiography':ti,ab OR 'm-mode echocardiography':ti,ab OR 'transthoracic echocardiography':ti,ab OR 'two-dimensional echocardiography':ti,ab OR ultrasound:ti,ab) AND [embase]/lim

(Lilacs = 02)
((Fetus OR Fetal* OR Fetus*) AND ("Global Longitudinal Strain" OR LV-GLS OR "Left Ventricular Global Longitudinal Strain" OR RV-GLS OR "Right Ventricular Global Longitudinal Strain" OR speckle-tracking OR "2D strain" OR "Longitudinal strain") AND ("reference values" OR "normal values" OR nomogram OR "reference range" OR "normal range") AND (echocardiography OR "2-D Echocardiography" OR "2D Echocardiography" OR "Cross-Sectional Echocardiography" OR "M-Mode Echocardiography" OR "Transthoracic Echocardiography" OR "Two-Dimensional Echocardiography" OR ultrasound))
