# Supplementary material for: Reference Ranges for Fetal Ventricular Global Longitudinal Strain (GLS) Using Bidimensional Speckle-Tracking Echocardiography: A Systematic Review
Source: J Clin Med. 2026 Aug 24;15(17):6536. doi: 10.3390/jcm15176536 (PMC13566355; doi:10.3390/jcm15176536)
Supplement: Supplementary file 1 [file jcm-15-06536-s001.zip › jcm-4423392-supplementary/S2_List_of_excluded_articles_with_reason.pdf]

S2 – List of excluded articles with reason.

**Reason for exclusion**	**Studies excluded from systematic review**
It was not a scientific article, or it was a review, case report, letter, study protocol, doctoral thesis, editorial, author index, supplementary material, or conference abstract.	(1, 19, 20, 21, 24, 25, 28, 29, 32, 37, 41, 43, 57, 58, 64, 67, 68, 70, 71)
Do not evaluate fetuses, including only neonates, children, or adults, or without results presented separately for the fetal population.	(13, 15, 22, 23, 26, 30, 47, 51, 55, 56)
The population was not normal fetuses, but rather had pathologies or congenital anomalies, or were twins. Pregnant women had comorbidities.	(2, 14, 27, 36, 39, 40, 42, 53, 59, 60, 72, 73)
They were not prospective observational studies (cross-sectional or cohort).	(5, 6, 7, 31, 50, 63, 65)
They did not have an explicit objective of establishing normal reference values (nominal curves, tables, or intervals).	(8, 11, 12, 17, 18, 46, 49)
They did not evaluate myocardial deformation by 2D speckle tracking (2D deformation) to obtain global longitudinal strain (GLS) measurements of the left and/or right ventricle, evaluating only conventional echocardiographic parameters or other types of strain without global longitudinal GLS.	(3, 4, 9, 10, 16, 33, 34, 35, 38, 52, 54, 61, 62, 66, 69)
They did not use fetal echocardiography/cardiac ultrasound as the primary imaging method or employed other non-echocardiographic methods.	(44, 45, 48)

1. The Thoracic and Cardiovascular Surgeon. 51st Annual Meeting German Society for Pediatric Cardiology. *Thorac Cardiovasc Surg*. 2026;74(1). doi:10.1055/s-00000085.
2. Altit G, Bhombal S, Chock VY, Tacy TA. Immediate postnatal ventricular performance is associated with mortality in hypoplastic left heart syndrome. *Pediatr Cardiol*. 2019;40(1):168-176. doi:10.1007/s00246-018-1974-9.
3. Anuwutnavin S, Russameecharoen K, Ruangvutilert P, Viboonchard S, Sklansky M, Devore GR. Assessment of the size and shape of the 4-chamber view and the right and left ventricles using fetal speckle tracking in normal fetuses at 17–24 gestational weeks. *Fetal Diagn Ther*. 2022;49(1-2):41-51. doi:10.1159/000521378.
4. Chelliah A, et al. Myocardial strain can be measured from first-trimester fetal echocardiography using velocity vector imaging. *Prenat Diagn*. 2016;36(5):483-488.
5. Cong J, Wang Z, Jin H, et al. Quantitative evaluation of longitudinal strain in layer-specific myocardium during normal pregnancy in China. *Cardiovasc Ultrasound*. 2016;14:45. doi:10.1186/s12947-016-0089-9.

6. Dahlbäck C, Gudmundsson S. Investigations on atrial function in fetuses with signs of impaired placental function. *Prenat Diagn*. 2015;35(6):605-611.

7. Dallaire F, Sarkola T. Growth of cardiovascular structures from the fetus to the young adult. *Adv Exp Med Biol*. 2018;1065:347-360. doi:10.1007/978-3-319-77932-4_22.

8. Devore GR, Klas B, Satou G, Sklansky M. Speckle tracking of the basal lateral and septal wall annular plane systolic excursion of the right and left ventricles of the fetal heart. *J Ultrasound Med*. 2019;38(5):1309-1318. doi:10.1002/jum.14811.

9. Devore GR, Klas B, Satou G, Sklansky M. Speckle tracking analysis to evaluate the size, shape, and function of the atrial chambers in normal fetuses at 20–40 weeks of gestation. *J Ultrasound Med*. 2022;41(8):2041-2057. doi:10.1002/jum.15888.

10. Devore GR, Klas B, Satou G, Sklansky M. Longitudinal annular systolic displacement compared to global strain in normal fetal hearts and those with cardiac abnormalities. *J Ultrasound Med*. 2018;37(5):1159-1171. doi:10.1002/jum.14454.

11. Devore GR, Polanco B, Satou G, Sklansky M. Two-dimensional speckle tracking of the fetal heart: a practical step-by-step approach for the fetal sonologist. *J Ultrasound Med*. 2016;35(8):1765-1781. doi:10.7863/ultra.15.08060.

12. Devore GR, Klas B, Satou G, Sklansky M. Evaluation of the right and left ventricles: an integrated approach measuring the area, length, and width of the chambers in normal fetuses. *Prenat Diagn*. 2017;37(12):1203-1212. doi:10.1002/pd.5166.

13. De Waal K, Phad N, Lakkundi A, Tan P. Post-transitional adaptation of the left heart in uncomplicated, very preterm infants. *Cardiol Young*. 2017;27(6):1167-1173. doi:10.1017/S1047951116002687.

14. Eckersley LG, et al. Quantitative assessment of left ventricular dysfunction in fetal Ebstein's anomaly and tricuspid valve dysplasia. *J Am Soc Echocardiogr*. 2019;32(12):1598-1607.

15. Elkiran O, Karakurt C, Kocak G, Karadag A. Tissue Doppler, strain, and strain rate measurements assessed by two-dimensional speckle-tracking echocardiography in healthy newborns and infants. *Cardiol Young*. 2014;24(2):201-211. doi:10.1017/S1047951112002284.

16. Elmstedt NN, Johnson JJ, Lind BB, et al. Reference values for fetal tissue velocity imaging and a new approach to evaluate fetal myocardial function. *Cardiovasc Ultrasound*. 2013;11:29. doi:10.1186/1476-7120-11-29.

17. Enzensberger C, Degenhardt J, Tenzer A, Doelle A, Axt-Fliedner R. First experience with three-dimensional speckle tracking in fetal echocardiography. *Ultraschall Med*. 2014;35(6):566-572. doi:10.1055/s-0034-1384882.

18. Enzensberger C, Rostock L, Graupner O, et al. Wall motion tracking in fetal echocardiography—application of low and high frame rates for strain analysis. *Echocardiography*. 2019;36(2):386-393. doi:10.1111/echo.14238.

19. Enzensberger C, et al. Three-dimensional speckle tracking in fetal echocardiography: a new technology in assessment of fetal heart function. *Ultraschall Med*. 2013;34(Suppl 1):PS6_05.

20. Enzensberger C, Tenzer A, Doelle A, Degenhardt J, et al. OP04.10: Assessment of area strain, twist, torsion and rotation in fetal echocardiography using three-

dimensional wall motion tracking technology. *Ultrasound Obstet Gynecol*. 2013;42(Suppl 1):59.

21. Enzensberger C, Tenzer A, Doelle A, et al. Three-dimensional speckle tracking in fetal echocardiography. *Z Geburtshilfe Neonatol*. 2013;217(Suppl 1):V11_2.

22. Eroglu N, Yoldas T, Bilgin B, et al. Intrauterine growth retardation is a risk factor for anthracycline toxicity. *Pediatr Hematol Oncol*. 2021;38(5):497-503. doi:10.1080/08880018.2021.1897717.

23. De Boode WP, Singh Y, Molnar Z, et al. Application of neonatologist performed echocardiography in the assessment and management of persistent pulmonary hypertension of the newborn. *Pediatr Res*. 2018;84(Suppl 1):68-77. doi:10.1038/s41390-018-0082-0.

24. Fratta F, Ricci C, Morelli C, et al. Prenatal prediction of aortic coarctation: 15 years of experience. In: *XLI Congresso Nazionale della Società Italiana di Cardiologia Pediatrica*; Oct 12–15, 2011; Bari, Italy.

25. Potential added value of bidimensional myocardial strain in prenatal diagnosis of aortic coarctation. *Cardiol Young*. 2012;22(Suppl 1):S3-S176. doi:10.1017/S1047951112000509.

26. Gardiner HM, Matsui H, Roughton M, et al. Cardiac function in 10-year-old twins following different fetal therapies for TTTS. *Ultrasound Obstet Gynecol*. 2014;43(6):652-657. doi:10.1002/uog.13279.

27. Germanakis I, Matsui H, Gardiner HM. Myocardial strain abnormalities in fetal congenital heart disease assessed by speckle tracking echocardiography. *Fetal Diagn Ther*. 2012;32(1-2):123-130. doi:10.1159/000334413.

28. Germanakis I. Supplementary material for myocardial strain abnormalities in fetal congenital heart disease assessed by speckle tracking echocardiography [supplementary material]. Figshare; 2017. doi:10.6084/m9.figshare.5123068.v1.

29. 56th Annual Meeting of the Association for European Paediatric and Congenital Cardiology (AEPC). *Cardiol Young*. 2023;33(Suppl 1):S1-S319. doi:10.1017/S1047951123001099.

30. Gziri MM, Hui W, Amant F, et al. Myocardial function in children after fetal chemotherapy exposure. *Eur J Pediatr*. 2013;172(2):163-170. doi:10.1007/s00431-012-1849-7.

31. Harbison AL, Pruetz JD, Ma S, et al. Evaluation of cardiac function in the recipient twin after TTTS using fetal speckle tracking. *Prenat Diagn*. 2021;41(1):136-144. doi:10.1002/pd.5835.

32. Harbison AL, et al. Regional cardiac remodeling following laser surgery for TTTS. *Am J Obstet Gynecol*. 2019;220(1):S561-S562.

33. Huntley ES, Hernandez-Andrade E, Soto E, et al. Reproducibility of fetal heart size, shape, and strain using speckle tracking. *Fetal Diagn Ther*. 2021;48(7):541-550. doi:10.1159/000517625.

34. Inamura N, Machida M, Nakamura T, et al. Fetal 24-segment sphericity index and ventricular fractional shortening at 12–20 weeks. *J Ultrasound Med*. 2024;43:1849-1855. doi:10.1002/jum.16516.

35. Ishii T, McElhinney DB, Harrild DM, et al. Circumferential and longitudinal ventricular strain in the normal human fetus. *J Am Soc Echocardiogr*. 2012;25(1):105-111. doi:10.1016/j.echo.2011.09.016.

36. Ishii T, Tworetzky W, Harrild DM, McElhinney DB. Left ventricular function and geometry in fetuses with severe tricuspid regurgitation. *Ultrasound Obstet Gynecol*. 2012;40(1):55-61. doi:10.1002/uog.10115.

37. Jones JE. *Speckle tracking as a tool for assessing foetal cardiac ventricular imbalance (STACVI).* Dissertation. Manchester Metropolitan University; 2022.

38. Köster HA, Hammer K, Braun J, et al. Comparison of strain measurements in fetal STE using Philips and TomTec. *J Perinat Med*. 2020;48(3):266-273. doi:10.1515/jpm-2019-0424.

39. Lee-Tannock A, Hay K, Gooi A, Kumar S. Longitudinal assessment of ventricular strain and annular plane excursion in diabetic pregnancies. *Eur J Obstet Gynecol Reprod Biol*. 2021;256:364-371. doi:10.1016/j.ejogrb.2020.11.057.

40. Lee W, Mack LM, Miremadi R, et al. Cardiac size, shape, and contractility in small-for-gestational-age fetuses. *J Ultrasound Med*. 2022;41(11):2703-2714. doi:10.1002/jum.15954.

41. Author index. *J Am Soc Echocardiogr*. 2013;26(6):B129-B152.

42. Liu B, Dardeer AM, Moody WE, et al. Normal values for right heart myocardial deformation by CMR feature tracking. *Int J Cardiol*. 2018;252:220-223. doi:10.1016/j.ijcard.2017.10.106.

43. Gonzalez R, Holmes W. Editorial for reference values for fetal cardiac dimensions and strain by CMR. *J Magn Reson Imaging*. 2023;60:375-376.

44. Minocha PK, Englund EK, Friesen RM, et al. Reference values for fetal cardiac dimensions and left ventricular strain by CMR. *J Magn Reson Imaging*. 2024;60(1):365-374. doi:10.1002/jmri.29077.

45. Minocha PK, Englund EK, Friesen RM, et al. Reference values for fetal cardiac dimensions and left ventricular strain by CMR. *J Magn Reson Imaging*. 2024;60(1):365-374. doi:10.1002/jmri.29077.

46. Nakata M, Nagasaki S. Two-dimensional tracking technique for assessment of fetal cardiac function. *Donald School J Ultrasound Obstet Gynecol*. 2021;15:254-258.

47. Nayak V, et al. Subclinical myocardial dysfunction among fetal growth restriction neonates. *J Matern Fetal Neonatal Med*. 2024;37(1):2392783.

48. Nichting TJ, et al. BEATS-study protocol. *BMC Pregnancy Childbirth*. 2021;21(1):524.

49. Nyrnes SA, Fadnes S, Wigen MS, et al. Blood speckle-tracking using high-frame-rate ultrasound in pediatric cardiology. *J Am Soc Echocardiogr*. 2020;33(4):493-503.e5. doi:10.1016/j.echo.2019.11.003.

50. Nyrnes SA, Garnæs KK, Salvesen Ø, et al. Cardiac function in newborns of obese women. *PLoS One*. 2018;13(6):e0197334. doi:10.1371/journal.pone.0197334.

51. Peček J, Koželj M, Lenasi H, Fister P. Right ventricular function in neonates during early postnatal period. *Pediatr Cardiol*. 2022;43(6):1327-1337. doi:10.1007/s00246-022-02855-7.

52. Sengupta SP, Bansal M, Hofstra L, Sengupta PP, Narula J. Gestational changes in left ventricular myocardial contractile function. *Int J Cardiovasc Imaging*. 2017;33(1):69-82. doi:10.1007/s10554-016-0977-y.

53. Sonaglioni A, Braga M, Villa MC, et al. Biventricular myocardial function in infants of gestational diabetic mothers. *Acta Diabetol*. 2022;59(9):1145-1156. doi:10.1007/s00592-022-01906-y.

54. Su XR, Wang AL, Tie HX, et al. Clinical application of the fetal HQ technique. *Echocardiography*. 2025;42(1):e70078. doi:10.1111/echo.70078.

55. Toma DA, Togănel R, Făgărășan A, et al. Interobserver agreement and reference ranges for neonatal strain. *Int J Environ Res Public Health*. 2022;19.

56. Toma D, Făgărășan A, Cerghit-Paler A, et al. Myocardial function in late preterm versus term neonates. *Front Pediatr*. 2024;12:1302383. doi:10.3389/fped.2024.1302383.

57. Van Oostrum NHM, De Vet CM, Van der Woude DAA, et al. Fetal strain and strain rate during pregnancy: a systematic review. *Eur J Obstet Gynecol Reprod Biol*. 2020;250:178-187. doi:10.1016/j.ejogrb.2020.04.002.

58. Van Oostrum NHM, Oei SG, Van Laar JOEH. Normal fetal cardiac deformation values: cohort study protocol. *BMC Pregnancy Childbirth*. 2019;19(1):524. doi:10.1186/s12884-019-2662-3.

59. Willruth AM, et al. Cardiac function in monochorionic diamniotic twins with TTTS assessed by speckle tracking. *Ultraschall Med*. 2013;34(2):162-168.

60. Yerlikaya-Schatten G, et al. Cardiac function in pregnant women with preeclampsia. *Front Cardiovasc Med*. 2024;11:1415727.

61. Zhao L, Wu P, Jiao X, et al. Characteristics and outcomes of fetal ventricular aneurysm and diverticulum. *Front Pediatr*. 2023;11:1165972. doi:10.3389/fped.2023.1165972.

62. Zhu C, et al. Reference ranges of cardiac size and morphology using 2D STE. *Fudan Univ J Med Sci*. 2024;51(1):41-49.

63. Zhu M, et al. Real-time 3D echocardiographic evaluation of fetal myocardial strain. *Echocardiography*. 2015;32(11):1697-1706.

64. Biancardi M. *Nomograma do strain miocárdio fetal na gestação.* Master's thesis. Instituto Fernandes Figueira (Fiocruz); 2019.

65. Clavero Adell M, Ayerza Casas A, Jiménez Montañés L, et al. Evolution of strain and strain rate throughout gestation in healthy fetuses. *Int J Cardiovasc Imaging*. 2020;36(1):59-66. doi:10.1007/s10554-019-01695-6.

66. Di Salvo G, Russo MG, Paladini D, et al. Two-dimensional strain to assess ventricular longitudinal function in normal fetuses. *Eur J Echocardiogr*. 2008;9(6):754-756. doi:10.1093/ejechocard/jen134.

67. Domínguez-Gallardo C, Ginjaume-García N, Ullmo J, et al. Supplementary material for gestational age-adjusted reference ranges for fetal LV strain. Figshare; 2022. doi:10.6084/m9.figshare.21324354.v1.

68. Ge S, Tao G, Zhan X, et al. Right ventricular strain during the 2nd and 3rd trimester. *Eur Heart J*. 2011;32(Suppl):122-123. doi:10.1093/eurheartj/ehr322.

69. Maskatia SA, Pignatelli RH, Ayres NA, et al. Fetal and neonatal diastolic myocardial strain rate. *J Am Soc Echocardiogr*. 2016;29(7):663-669. doi:10.1016/j.echo.2016.02.017.

70. Meister M, Graupner O, Gotte M, et al. Speckle tracking in fetal echocardiography. *Thorac Cardiovasc Surg*. 2019;67(8):e1-e8. doi:10.1055/s-0039-1679072.

71. Meister M, Graupner O, Götte M, et al. Speckle tracking in fetal echocardiography. *Geburtshilfe Frauenheilkd*. 2018;78(10):1014-1021. doi:10.1055/s-0038-1671437.

72. Willruth AM, Geipel A, Berg C, et al. LV strain, strain rate and velocity using feature tracking. *Ultraschall Med*. 2012;33(7):E293-E298. doi:10.1055/s-0029-1246029.

73. Willruth AM, Geipel AK, Fimmers R, Gembruch UG. RV strain and impact of gestational age using feature tracking. *Ultrasound Obstet Gynecol*. 2011;37(2):143-149. doi:10.1002/uog.7719.
