# Supplementary material for: Reference Ranges for Fetal Ventricular Global Longitudinal Strain (GLS) Using Bidimensional Speckle-Tracking Echocardiography: A Systematic Review
Source: J Clin Med. 2026 Aug 24;15(17):6536. doi: 10.3390/jcm15176536 (PMC13566355; doi:10.3390/jcm15176536)
Supplement: Supplementary file 1 [file jcm-15-06536-s001.zip › jcm-4423392-supplementary/S3_Methodological_quality_criteria__Extracted_and_adapted_from_Oros_et_al.pdf]

S3 – Methodological quality criteria. Extracted and adapted from Oros et al. Extracted and reproduced from Table 1 – Methodological quality criteria for study design and for reporting and statistical methods in studies presenting reference ranges for fetal Doppler parameters

Domain	***Low risk of bias 1***	***High risk of bias 0***
Study design		
1.01 Study design	Clearly described as either cross-sectional or longitudinal	Not reported / Mixture of cross-sectional and longitudinal data
1.02 Population	Women reported as coming from population of low risk of pregnancy complications	Women from unselected population; or seleted; or at high risk of prebnancy complications, or not reported
1.03 Prospective data collection	Prospective study and ultrasound data collected specifically for purpose of constructing charts of fetal Doppler	Retrospective study, data not collected specifically for purpose of constructing chartsof fetal Doppler, or unclear (e.g. use of routinely collected data)
1.04 Specific scan	Specific scan for research purposes	Routine scan in context of pregnancy assessment
1.05 Sample size	*A-priori* determination or calculation of sample size and justification	Lack of *a-priori* sample size determination or calculation and justification
1.06 Recruitment period	Reported	Not reported
1.07 Consecutive enrolment	Consecutively included patients	Did not include patients consecutively
1.08 Inclusion/exclusion criteria	Made clear that women at high risk of pregnancy complications were not included and that women with abnormal outcome were excluded, i.e an effort was made to include as normal an outcome as possible	Study population included both low- and high-risk pregnancies, or women with abnormal outcome were not excluded
1.09 Method of dating pregnancy	Clearly described known LMP and sonogram before 14 weeks' gestation	Not described clearly
1.10 Multicenter study	Study performed with more than one center collaborating	Performed at only one hospital

Domain	*Low risk of bias 1*	*High risk of bias 0*
2 Reporting and statistical methods		
2.01 Perinatal outcome	Collected and reported prospectively	Not reported
2.02 Gestational age range	Reported	Not reported
2.03 Ultrasound machines and probe type used	Clearly specified	Not clearly specified
2.04 Reported sonographers	Number of sonographers reported	Not clearly specified
2.05 Sonographer experien ce/type of examiner reported (fetologist/ cardiologist)	Experienced or specifically trained sonographers clearly reported	Not clearly specified
2.06 Blinded measurements	Sonographers were blinded	Not clearly specified
2.07 Ultrasound quality control measures	Should include: assessment of intraobserver variability; assess ment of interobserver variability; image review; image scoring; image storage characteristics	Does not contain quality control measures
2.08 Protocol	Study described sufficient and unambiguous details of measure ment techniques used for fetal Doppler parameters	Not described sufficiently and unambiguous details of measurement
2.09 Number of measure ments taken for each variable	At least three measures per fetus per scan	Single measure or not reported
2.10 Angle correction	Clearly specified	Not clearly specified
2.11 Statistical methods	Clearly described and identified	Not clearly described and identified
2.12 Report of mean and SD of each measurement and sam ple size for each week of gestation	Presented in a table or clearly described	Not presented in a table or not clearly described
2.13 Report of regression equations for mean (and SD if relevant) for each measure ment (when applicable)	Reported	Not reported
2.14 Scatter diagram	Study included Doppler chart with mean and SD or centiles (at least 5th, 50th and 95th centiles)	Doppler charts not included

Source: Oros, D., Ruiz-Martinez, S., Urias, E.,Conde-Agudelo, A.,Villar, J.,Fabre, E., Papageorghiou, A. T. Reference ranges for Doppler indices of umbilical and fetal middle cerebral arteries and cerebroplacental ratio: systematic review. Ultrasound Obstet Gynecol 2019; 53: 454–464 https://doi.org/10.1002/uog.20102
